# Brain functional topology differs by sex in cognitively normal older adults

**DOI:** 10.1093/texcom/tgac023

**Published:** 2022-06-27

**Authors:** Zhengshi Yang, Filippo Cieri, Jefferson W Kinney, Jeffrey L Cummings, Dietmar Cordes, Jessica Z K Caldwell, for the Alzheimer’s Disease Neuroimaging Initiative

**Affiliations:** Department of Neurology, Cleveland Clinic Lou Ruvo Center for Brain Health, 888 W Bonneville Ave, Las Vegas, NV 89106, United States; Department of Brain Health, University of Nevada, Mail Stop: 4022; 4505 S. Maryland Pkwy. Room 1172, Las Vegas, NV 89154, United States; Department of Neurology, Cleveland Clinic Lou Ruvo Center for Brain Health, 888 W Bonneville Ave, Las Vegas, NV 89106, United States; Department of Brain Health, University of Nevada, Mail Stop: 4022; 4505 S. Maryland Pkwy. Room 1172, Las Vegas, NV 89154, United States; Chambers-Grundy Center for Transformative Neuroscience, University of Nevada, Box 454022, 4505 S. Maryland Pkwy, Las Vegas, NV 89154-4022, United States; Department of Brain Health, University of Nevada, Mail Stop: 4022; 4505 S. Maryland Pkwy. Room 1172, Las Vegas, NV 89154, United States; Chambers-Grundy Center for Transformative Neuroscience, University of Nevada, Box 454022, 4505 S. Maryland Pkwy, Las Vegas, NV 89154-4022, United States; Department of Neurology, Cleveland Clinic Lou Ruvo Center for Brain Health, 888 W Bonneville Ave, Las Vegas, NV 89106, United States; Department of Brain Health, University of Nevada, Mail Stop: 4022; 4505 S. Maryland Pkwy. Room 1172, Las Vegas, NV 89154, United States; Department of Psychology and Neuroscience, University of Colorado, 3100 Marine St., Boulder, CO 80309, United States; Department of Neurology, Cleveland Clinic Lou Ruvo Center for Brain Health, 888 W Bonneville Ave, Las Vegas, NV 89106, United States

**Keywords:** brain functional topology, functional MRI, graph theory, sex difference

## Abstract

**Introduction:**

Late onset Alzheimer’s disease (AD) is the most common form of dementia, in which almost 70% of patients are women.

**Hypothesis:**

We hypothesized that women show worse global FC metrics compared to men, and further hypothesized a sex-specific positive correlation between FC metrics and cognitive scores in women.

**Methods:**

We studied cognitively healthy individuals from the Alzheimer’s Disease Neuroimaging Initiative cohort, with resting-state functional Magnetic Resonance Imaging. Metrics derived from graph theoretical analysis and functional connectomics were used to assess the global/regional sex differences in terms of functional integration and segregation, considering the amyloid status and the contributions of APOE E4. Linear mixed effect models with covariates (education, handedness, presence of apolipoprotein [APOE] E4 and intra-subject effect) were utilized to evaluate sex differences. The associations of verbal learning and memory abilities with topological network properties were assessed.

**Result:**

Women had a significantly lower magnitude of the global and regional functional network metrics compared to men. Exploratory association analysis showed that higher global clustering coefficient was associated with lower percent forgetting in women and worse cognitive scores in men.

**Conclusion:**

Women overall show lower magnitude on measures of resting state functional network topology and connectivity. This factor can play a role in their different vulnerability to AD.

**Significance statement:**

Two thirds of AD patients are women but the reasons for these sex difference are not well understood. When this late onset form dementia arises is too late to understand the potential causes of this sex disparities. Studies on cognitively healthy elderly population are a fundamental approach to explore in depth this different vulnerability to the most common form of dementia, currently affecting 6.2 million Americans aged 65 and older are, which means that >1 in 9 people (11.3%) 65 and older are affected by AD. Approaches such as resting-state functional network topology and connectivity may play a key role in understanding and elucidate sex-dependent differences relevant to late-onset dementia syndromes.

## Introduction

Late onset Alzheimer’s disease (AD) is the most common form of dementia, in which age and sex, are the major risk factors. Almost 70% of AD patients are women (Alzheimer’s Association 2021), but the reasons for these sex disparities are not clear. One potentially important avenue of inquiry is better understanding baseline sex differences in cognitively healthy aging, with respect to brain function (Biswal et al. 2010; Ritchie et al. 2018; Weis et al. 2020), cognition (Ceci et al. 2009; Miller and Halpern 2014), and their interactions. This knowledge would provide answers to whether sex differences observed in AD are features of neurodegeneration, or are consequences of sex-specific neurocognitive aging, which in turn may inform studies of sex-specific risk for AD-related cognitive decline.

The majority of functional magnetic resonance imaging (fMRI) studies treat sex as a covariate of limited interest, and the role of sex in brain function has not been intensively investigated.

The healthy brain is considered a complex dynamic system composed of networks with multiple spatial and time scales, modular structure, where a balance is necessary between local segregation and global integration (Geschwind, 1965; Tononi et al. 1994; Delbeuck et al. 2003; Cieri et al. 2021a; Yang et al. 2022). Considering AD a disconnection syndrome (Contreras et al. 2019; Cieri et al. 2021b) to which women are more vulnerable, resting-state functional network topology and connectivity is an important approach to explore resting-state fMRI (rs-fMRI) connectivity patterns. It is crucial applying these studies from the cognitive healthy stage, in order to understand whether potential differences between men and women are present before the pathological condition.

A review on studies in children and young adults showed more between-module connectivity in men, and more within-module connectivity in women (Gur and Gur 2017). Across the lifespan healthy women have shown higher cortical functional connectivity (FC) in the left hemisphere, whereas higher values were found in the right hemisphere of men (Gong et al. 2009). One of the largest studies in the field (Ritchie et al. 2018) has shown that the strength of FC between sensorimotor, visual, and rostral lateral prefrontal areas was higher in men compared with women. On the other hand, the strength of FC within the default network (DN) was higher in women than men.

Recently, we have shown that cognitively healthy individuals, compared with individuals with mild cognitive impairment (MCI) and AD, had the most substantial sex differences in 5 global network metrics. The comparison between women and men have specifically shown that cognitively healthy women had significantly lower degree centrality, global efficiency, local efficiency, clustering coefficient, and significantly higher path length, compared with the latter (Cieri et al. 2021b). Moreover, better FC metrics were associated with better verbal learning scores, only in women. These findings suggest that sex plays a role in brain functional network topology and cognition in cognitively healthy older adults. However, that study did not explore the spatial specificity of our findings or effects of additional variables such as presence of APOE E4 allele and brain amyloid.

In the present research, we investigated sex differences in global and regional functional topological network properties and regional FC in a larger sample of fMRI sessions from cognitively healthy individuals 80 years old or younger. We used graph theoretical analysis and functional connectomics to derive network metrics for quantifying the connectivity strength, functional integration, and segregation. Then we employed LME models with covariates (education, handedness, amyloid status, APOE4 carrier status, and intra-subject effect) to evaluate sex differences. Based on our recent results (Cieri et al. 2021b), we hypothesized that women would show worse global FC metrics compared with men, and further hypothesized a sex-specific positive correlation between FC metrics and cognitive scores in women. Since these subjects are cognitively healthy we did not expect an association with APOE4 or amyloid positivity.

## Methods

### Participants

Data used in this study were obtained from the Alzheimer’s Disease Neuroimaging Initiative (ADNI) database. The study was approved by each participating ADNI site’s local Institutional Review Boards, as documented on the ADNI website. All participants gave written, informed consent. The sponsors for ADNI are listed in the Funding section. All subjects enrolled in this study were required to have 3.0-Tesla resting-state fMRI and T1-weighted structural MRI data available, and diagnosed as cognitively normal at the corresponding visit. Women had significantly better 11-item Alzheimer’s Disease Assessment Scale–Cognitive subscale (ADAS-Cog, 70-point scale) scores than men (*P* = 0.001) and showed a trend toward younger age (*P* = 0.11). As such, we excluded participants with age > 80 years or ADAS-Cog > 10 to achieve age- and cognition-matched woman and man participants. Seventy-seven fMRI sessions from 48 men (average 1.6 sessions per subject, age 72.2 ± 4.3 years, years of education 17.7 ± 2.2, and ADAS-Cog 5.7 ± 2.3) and 130 fMRI sessions from 74 women (average 1.8 sessions per subject, age 72.6 ± 4.5 years, years of education 16.3 ± 2.3, and ADAS-Cog 5.5 ± 2.2) were included in this study (Table 1).

**Table 1:** Demographic characteristics of women and men with normal cognition.

fMRI sessions (*n* = 207)	Men (*n* = 77)	Women (*n* = 130)	*P* value
Subjects	48	74	
AGE	72.2 ± 4.3	72.6 ± 4.5	0.53
Handedness [R/L]^a^	43/5	63/11	0.48
Education	17.7 ± 2.2	16.3 ± 2.3	3.5E-05
APOE4 [+/−]^a^	12/36	27/47	0.18
Amyloid status [+/−]^a^^b^	25/43	44/72	0.87
*CDRSB*	*0.1 ± 0.3*	*0.1 ± 0.2*	*0.95*
*ADAS-Cog [0–70]*	*5.7 ± 2.3*	*5.5 ± 2.2*	*0.63*

*Note:* Age, ADAS-Cog, and Aβ status are summarized over fMRI sessions; handedness, education, APOE4 are summarized over subjects. Two-sample *t*-test was carried out if not specified.

^a^Chi-square test statistic is used.

^b^Only some of the fMRI sessions have Amyloid PET scans within 1 year of fMRI sessions.

### Amyloid status

Considering that florbetapir amyloid positron emission tomography (PET) scans could be a few years away from the MRI scans and amyloid status could be altered in this period, florbetapir PET data were required to be collected within 1 year of MRI scan and the amyloid status for each fMRI session instead of each participant were determined. We extracted the composite standard uptake value ratio (SUVR) to determine the amyloid status by following the ADNI PET analysis pipeline. Following ADNI florbetapir PET processing method, the fMRI sessions with composite SUVR above 1.11 in florbetapir PET scans were defined as amyloid positive. The sessions with amyloid burden below the threshold were labeled as amyloid negative. 115 fMRI sessions (43 from men and 72 from women) were determined to be amyloid negative, and 69 fMRI sessions (25 from men and 44 from women) were amyloid positive with the amyloid status of 23 fMRI sessions (9 from men and 14 from women) unknown.

### Clinical and cognitive measures

Clinical dementia staging and neuropsychological tests were completed at each visit. Measures compiled in this study included the ADAS-Cog, Clinical Dementia Rating-sum of boxes (CDR-SB), Montreal Cognitive Assessment (MoCA), Trail Making Test-B (TMTB), and Rey Auditory Verbal Learning Test (RAVLT) learning, immediate recall, delayed recall, and percent forgetting.

### MRI acquisition and analysis

The T1-weighted magnetization-prepared rapid acquisition gradient-echo MR images were collected with a 24-cm field of view and a resolution of 256 × 256 × 170 to yield a 1 × 1 × 1.2-mm^3^ voxel size. The resting-state fMRI data were acquired from echo-planar imaging sequence with TR/TE = 3,000/30 ms, flip angle = 80°, 48 slices, spatial resolution = 3.3 × 3.3 × 3.3 mm^3^, and imaging matrix = 64 × 64.

#### fMRI preprocessing and denoising

The raw fMRI data were first processed with slice-timing correction and rigid-body realignment of all fMRI volumes to mean fMRI volumes using SPM12 (https://www.fil.ion.ucl.ac.uk/spm/). The first 5 volumes of fMRI data were discarded to avoid data with unsaturated T1 signals. The mean fMRI volumes were coregistered to the native T1 structural image and the T1 image was spatially normalized to MNI152 standard space. The transformation information from coregistration and space normalization steps were applied on each fMRI volume separately to transform fMRI data to the template space. Instead of using traditional nuisance regression techniques to de-noise fMRI data, an artificial intelligence technique was applied to remove the noise in each fMRI session separately (Yang et al. 2020).

This pipeline was conducted without any demographic/diagnostic information about the subject, thus it does not bias the post-processing analysis. Previous studies (Yang et al. 2020; Cieri et al. 2021b) demonstrated the improved statistical power of this technique over traditional de-noising strategies in identifying disrupted brain topology in subjects with AD.

#### FC network

Ninety-four cortical and subcortical regions in the cerebrum from the revised automated anatomical labelling [AAL] atlas (Rolls et al. 2015) were used in our analysis. The regional time series was defined as the mean time series over all voxels in each region. We then calculated Pearson’s correlation between regions followed by Fisher *r*-to-*z* transformation to construct FC network.

#### Global brain and regional network analysis

Graph theoretical analyses were then applied on the weighted FC maps to derive global and regional network metrics with sparsity level varying from 0.05 to 0.5 with increment of 0.01 (Wang et al. 2015). A detailed description of these network metrics is provided in Table 2. Nodal strength, nodal efficiency and clustering coefficient were assessed to characterize the functional topological organization of each brain region. The mean values of these 3 network metrics over 94 regions represent the global network topology of the whole brain. The global and regional network metrics integrated over all sparsity levels were the values used in the statistical analysis. We rescaled each network metric separately to similar range by converting the original values to their corresponding *z*-scores across all participants, which has no influence on the significance of the group difference between women and men.

**Table 2:** Global and regional network metrics assessed in this study.

*N* is the nodes (brain regions) defined by the AAL atlas and *n* is the number of nodes. ${w}_{ij}$ is the FC strength between node *i* and node *j*. Node *i* and node *j* are neighbors if the connectivity ${w}_{ij}$ surpasses the threshold. For each FC network at each sparsity level, the following global and nodal metrics were calculated.			
**Network characteristics**	**Nodal metrics**	**Global metrics**	**Equation**
**Strength** In the weighted, undirected network, strength reflects how strongly a node is connected with other nodes in the network.	**Nodal strength** Defined as the summation of weights from edges connected to a node.	**Connectivity strength** Average of nodal strength over all nodes	Nodal strength: ${k}_i={\sum}_{j\in N}{w}_{ij}$ Connectivity strength: $k={\sum}_{i\in N}{k}_i$
**Integration** Measures the efficiency of the parallel information transfer in the network.	**Nodal efficiency** The average of the inverse shortest path length between nodes. ${d}_{ij}$ denotes the shortest path length between node *i* and node *j*.	**Global efficiency** Average of nodal efficiency over all nodes.	Nodal efficiency: ${E}_i={\sum}_{j\in N,j\ne i}\frac{d_{ij}^{-1}}{n-1}$ Global efficiency: $E=\frac{1}{n}{\sum}_{i\in N}{E}_i$
**Segregation** Describes the likelihood that neighbors of a node are connected to each other and hence describes the tendency of the nodes to form local clusters.	**Nodal clustering coefficient** The proportion of a node’s neighbors that are also the neighbors of each other. ${t}_i$ denotes the geometric mean of triangles around node *i*, ${t}_i=\frac{1}{2}{\sum}_{j,h\in N}{\Big({w}_{ij}{w}_{jh}{w}_{hi}\Big)}^{1/3}$	**Clustering coefficient** Average of nodal clustering coefficient over all nodes.	Nodal clustering coefficient: ${C}_i=\frac{2{t}_i}{k_i\Big({k}_i-1\Big)}$ Clustering coefficient: $C=\frac{1}{n}{\sum}_{i\in N}{C}_i$

### Statistical analysis

LME model was utilized to assess the sex difference of global and regional network metrics, where the within-subject variance was modeled as a random effect grouped by individual subject and the confounding variables such as age, education, handedness, and APOE status (0: no E4 alleles and 1: 1 or 2 E4 copies) were modeled as fixed effects together with sex (network metrics ~ sex + age + handedness + education + APOE + (1|subject)). The same statistical analysis used for network metrics was performed with inter-regional FC. From the LME model, we extracted the adjusted network metrics and interregional connectivity after correcting for the influence of confounding factors and intra-subject effect. Then 2-sample *t*-statistic was applied to evaluate the difference between women and men. In order to summarize the regional network properties in different brain areas, 94 regions of interest were grouped into 6 anatomical macro-areas: prefrontal lobe; other parts of frontal lobe; occipital lobe; temporal lobe; parietal lobe; and central structures (Supplementary Table 1).

For both global and regional network analysis, the significance levels were reported after Bonferroni correction over multiple comparisons. As to inter-regional connectivity analysis, the commonly used network-based statistics (NBS; Zalesky et al. 2010) were then applied to deal with multiple comparisons problem. The primary significance threshold was set to 0.005 in NBS and the clusters with family-wise error corrected *P* values <0.05 were reported in the result, by running a nonparametric permutation analysis [10,000 permutations].

#### Correlation analysis

Exploratory correlation analysis tested the association of global network metrics with clinical and cognitive measures by conducting linear regression analysis for women and men separately. The *t*-test was used to compare the significance level of slope difference between women and men.

#### Assessment of APOE and amyloid effect

In order to assess whether APOE and amyloid status contributed to differences observed in this study, 2-way analysis of variance (ANOVA) was conducted to analyze their main effect on global network metrics along with their interaction effect with sex.

## Results

### Sex differences of global and regional network metrics

**Fig. 1:**
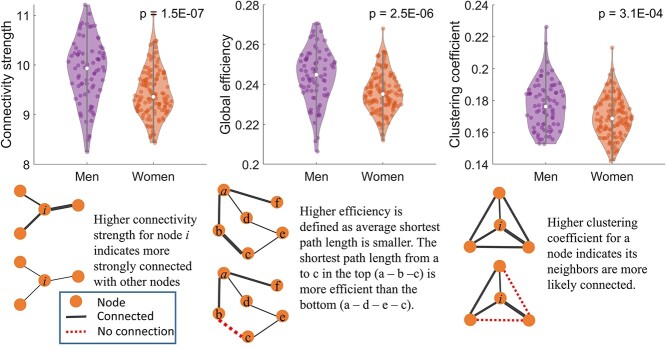
A comparison of global network metrics between women and men with normal cognition. The values presented in the figure were adjusted for confounding factors using LME model.

With the global network metrics, men showed significantly higher connectivity strength (mean ± SD: men 9.861 ± 0.642, women 9.431 ± 0.487; *P* = 1.5E−7), global efficiency (mean ± SD: men 0.244 ± 0.014, women 0.236 ± 0.010; *P* = 2.5E−6), and clustering coefficient (mean ± SD: men 0.177 ± 0.015, women 0.170 ± 0.013; *P* = 3.1E−4) than women (Fig. 1).

**Fig. 2:**
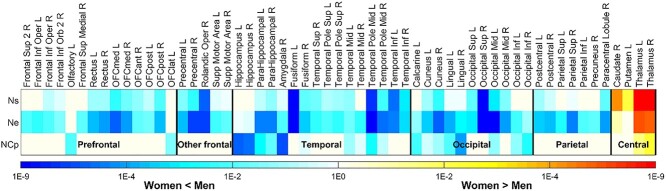
A comparison of regional network metrics between women and men with normal cognition, including nodal strength [Ns], nodal efficiency [Ne], and nodal clustering coefficient [NCp]. The significance levels of sex difference, characterized with *P* value after Bonferroni correction over number of brain regions. Only the regions having significant sex difference [Bonferroni-corrected *P* < 0.05] for at least one network metric are shown.

The analysis of regional network properties in women compared with men showed significant sex differences (Bonferroni-corrected *P* < 0.05) for nodal strength (23 regions), clustering efficient (14 regions), and nodal efficiency (49 regions; see Fig. 2 and Supplementary Table 2). Consistent with the global network metrics, the majority of brain regions showed lower magnitude in women than men across 3 regional network metrics. Temporal and occipital lobes were prominently involved with lower values in women than men for all 3 network metrics. Parietal and frontal lobes (including prefrontal and other frontal lobe regions) substantially contributed to the lower nodal efficiency in women than men and contributed to weaker nodal strength in women than men. In contrast, clustering coefficients in women differed little from men in frontal and parietal lobes. Lower nodal clustering coefficients in women than men were mainly located in temporal and occipital lobes. The significance of the difference for each region is shown in the Supplementary Table 2.

Higher regional network metrics in women were observed only at the dorsal striatum, specifically right caudate (*P* = 5.4E−5) and left putamen (*P* = 6.4E−3), where women had significantly higher nodal strength, compared with men. In addition, at the level of bilateral thalamus, women had significantly higher nodal strength (left *P* = 1.90E−10, right *P* = 1.45E−10), nodal efficiency (left *P* = 1.25E−5, right *P* = 1.35E−5), and clustering coefficient (left *P* = 0.045, right *P* = 4.08E−3) compared with men.

### Sex differences of inter-regional connectivity

**Fig. 3:**
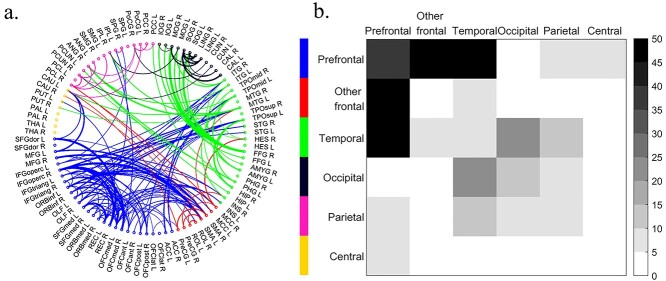
Inter-regional FC analysis. (a) The cluster detected to have significantly lower FC in women than men [NBS cluster-wise corrected significance level, *P* = 0.0012]. The marker colors represent the 6 brain macroareas which brain regions are assigned to and the line width of each connections denotes the significance of the difference between women and men. Please refer to the Supplementary Table 1 for the abbreviations in the figure. (b) The number of connections within/between macroareas showing lower FC in women in the cluster.

**Fig. 4:**
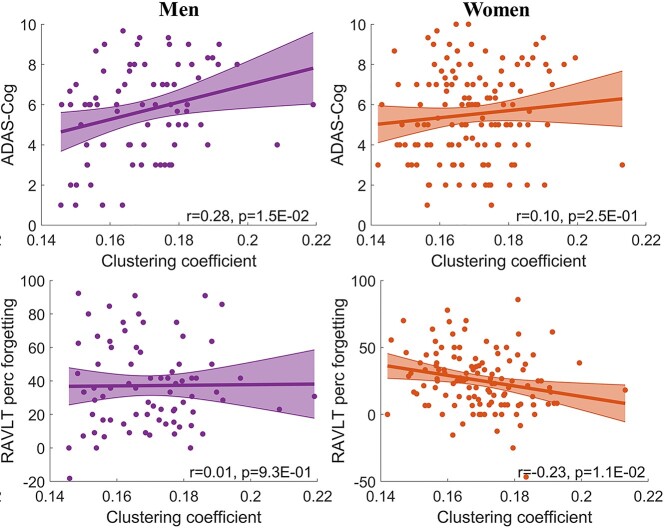
Exploratory correlation analysis between global network metrics and neuropsychological scores. The scatter plots and the linear fitting lines with 95% confidence interval were shown in the figure. Pearson’s correlation [*r*] and the significance of the correlation [*P*; uncorrected] were marked. The association analysis was conducted with all participants, men only and women only separately. Only clustering coefficient was observed to be associated with neuropsychological scores. Connectivity strength and global efficiency were not associated with neuropsychological scores.

In the inter-regional connectivity analysis, NBS detected one cluster showing significantly lower FC in women than men (NBS cluster-wise corrected *P* = 0.0012; Fig. 3), with the connectivity mainly within the prefrontal area, between prefrontal and temporal lobe, and between prefrontal, and other parts of frontal lobe. Fig. 3a demonstrated the connections in the cluster, and Fig. 4b showed the number of connections in the significant cluster having lower FC in women than men. No cluster was found to have higher FC in women than men.

### APOE, amyloid status and their interaction with sex

Two-way ANOVA revealed that APOE4 carriers and non-E4 carriers did not show differences on global network metrics (*P*_APOE_ > 0.7; Supplementary Fig. 1 top), and there was no difference between amyloid positive and amyloid negative participants (*P*_Amy_ > 0.8; Supplementary Fig. 1 bottom). Neither APOE nor amyloid status had significant interaction effects with sex (*P*_int_ > 0.2).

### Exploratory analysis of clinical correlation

In the exploratory association analysis between global network metrics and neuropsychological scores, only the clustering coefficient showed significant associations in the association analysis (uncorrected *P* < 0.05; see Fig. 4). Men showed worse ADAS-Cog score associated with higher clustering coefficient (*r* = 0.28, *P* = 0.015 uncorrected) but no significant association was observed with women. The slope difference between women and men was not significant. Higher clustering coefficient in women correlated with lower RAVLT percent forgetting (*r* = −0.23, *P* = 0.011 uncorrected) but men did not show an association between RAVLT percent forgetting score and clustering coefficient, a significant slope difference between women and men was observed (*P* = 0.046 uncorrected). No other neuropsychological scores had significant association with global network metrics. The associations of regional clustering coefficients with ADAS-Cog or RAVLT percent forgetting score were included in the Supplementary Fig. 2.

## Discussion

This study showed that among cognitively healthy elderly participants, women showed lower magnitude global clustering coefficient, connectivity strength, and global efficiency, compared with men. From a regional perspective, men overall had higher regional network values across all 3 metrics, especially at the temporal and occipital lobes, which is in line with higher global network values in men. Women had higher nodal strength at right caudate and left putamen compared with men. Also, at the level of bilateral thalamus, women showed significantly higher values. Higher global clustering coefficient was associated with lower RAVLT percent forgetting in women; in contrast, higher clustering coefficient related to worse ADAS-Cog scores in men. Overall, these findings confirm sex effects in brain function in healthy aging and suggest less efficient functional communication in cognitively healthy women compared with men. These observations suggest that the FC changes contribute to women’s higher vulnerability to AD. Moreover, this functional difference was independent of effects of APOE genotype and amyloid, indicating a potentially separate non-AD specific vulnerability.

Brain functional complexity seems to decrease with physiological and especially cognitively pathological aging (Cieri et al. 2021a), where AD in particular can be seen as a disconnection syndrome based on network deterioration (Contreras et al. 2019). Investigation of brain FC and complexity, in terms of network integration and segregation, can play a key role in the identification of early biomarkers to predict the evolution of healthy cognitive aging and/or pathology, which would be important for early diagnosis and intervention (Griffa et al. 2013; Griffa and Van Den Heuvel 2018).

In our study, women show lower global values in all the 3 metrics: clustering coefficient, connectivity strength, and global efficiency. Clustering coefficient is a well-known measure quantifying the small-worldness (van den Heuvel and Sporns 2013) of a specific network. A small-world network is a network with a large clustering coefficient and a small shortest path length between 2 nodes on average (Watts and Strogatz 1998; Bullmore and Sporns 2009; Masuda et al. 2018) and the loss of these features are typical of cognitively pathological aging, such as that seen in AD (Supekar et al. 2008; Brier et al. 2012) and MCI, as well as healthy cognitive aging, compared with younger participants (Grady et al. 2016).

Clustering coefficient is a direct measure of segregation, measuring the degree to which a network is organized into local specialized regions (Watts and Strogatz 1998; Bullmore and Sporns 2009; Griffa et al. 2013). Our results confirm a globally less segregated brain in cognitively healthy women compared with men.

Global connectivity strength is a measure of how strongly a node is connected with other nodes, through edges (van den Heuvel and Sporns 2011). Our findings demonstrate a global lower magnitude in women compared with men, similar to studies in AD subjects, where AD patients exhibited decreased node strength, local clustering coefficient, and local efficiency compared with cognitively healthy controls (Hallett et al. 2020). This result confirms our previous study, in which cognitively healthy women have shown a topological pattern closer to pathological cognitive aging (Cieri et al. 2021b).

Global efficiency is another measure of functional integration, described as the inverse of the average characteristic path length between all nodes in the network (Latora and Marchiori 2001), measuring the efficiency of distant information transfer within a network. Again, less global efficiency is a typical result not only in AD (Stam et al. 2007; Supekar et al. 2008), but also in MCI (Wang et al. 2013) and in cognitively healthy elderly individuals (Meunier et al. 2009; Sala-Llonch et al. 2012). Achard and Bullmore (2007) have shown that cognitively healthy elderly individuals have a less efficient global network, whereas Meunier et al. (2009) found a global reassessment of the modular organization in the healthy elderly brain, compared with younger brain. These results are consistent with our recent study (Cieri et al. 2021b). Women seem to lose more modularity, more small-word functionality, compared with men. The modularity describes a fundamental rule of biological systems, in which integration within subsystems allows efficient local processing (Simon 2012; Stevens et al. 2012). Small-world networks balance (Cieri et al. 2021a) between integration and segregation, with some densely interconnected groups of nodes and some long-range connections that allow fast information transferability (Watts and Strogatz 1998).

At the regional level, clustering coefficients in women were similar to those of men in frontal and parietal lobes. On the other hand, temporal and occipital lobes were prominently involved with lower values in women than men for all 3 network metrics. Temporal and occipital lobes are both part of the DN (Raichle et al. 2001), also called “task negative network” that is active and synchronized when the individual is not engaged in any external cognitive demanding task in the scanner during the resting-state (Fox and Raichle 2007; Christoff et al. 2016; Cieri and Esposito 2018, 2019; Cieri et al. 2020). This network includes the posterior cingulate cortex/precuneus, medial prefrontal cortex, inferior parietal lobules, lateral temporal cortices, and hippocampus (Raichle et al. 2001; Buckner and Carroll 2007). DN is of fundamental importance for cognition because its activity during rest has a key role for memory consolidation (Fox and Raichle 2007). Structures and function of this network are impaired in pathological aging (Cieri and Esposito 2018; Esposito et al. 2018), therefore it becomes essential to explore these features in cognitively healthy aging, before neurodegeneration occurs. There are data that show an overlap between DN hubs and anatomical patterns of amyloid deposits in AD, making this network an area of interest in physiological and pathological aging (Grady 2012; Cieri and Esposito 2018; Cieri et al. 2020). It is important to stress that connectivity in the DN is reduced not only in pathological neuroaging, but also in elderly subjects when compared with younger participants (Grady 2012). In this sense women seem to show an “older functional connectivity pattern” at least from the perspective of graph theory metrics. Aging impacts the segregation within networks and the integration of different networks (Geerligs et al. 2015), and women’s brain functional organization from this perspective, shows a “more aged functional neuro-configuration.”

Higher clustering coefficient is associated with better cognitive performance (lower RAVLT percent forgetting) only in women, but not in men, where conversely better clustering coefficient is associated with worse cognitive performance (ADAS-Cog). In other words when the global clustering coefficient increases in women, cognitive scores increase with it, whereas in men the opposite is observed. Some studies have shown an increase of clustering coefficient in pathological aging brains, such as MCI and AD (Yao et al. 2010), but in our case the sample is composed by cognitively healthy subjects, where higher global clustering coefficient is associated with better cognitive performance. In our previous study (Cieri et al. 2021b), we found a similar correlation only in cognitively healthy women—not in men—between learning score and another measure of integration (degree centrality).

When we look at the regional level of FC, we found higher regional network metrics in men across all our 3 metrics, consistent with our global values. Frontal and parietal lobes especially contributed to the lower nodal efficiency in women compared with men and conferred to women weaker nodal strength. In contrast, clustering coefficients in women differed little from men in frontal and parietal lobes. Importantly, compared with men, women have lower nodal clustering coefficient primarily at the level of temporal and occipital lobes.

It is possible that the global FC degenerates earlier in women and this can have a role in their higher vulnerability to neurodegeneration. This could be influential especially when looking at the regional level contributing to the global effect. Temporal and occipital lobes were especially involved in global effects, with lower values in women than men for all 3 metrics.

The only regions where women showed higher values are the caudate and left putamen and significantly higher nodal strength, nodal efficiency, and clustering coefficient at the level of bilateral thalamus, compared with men. These results are consistent with observations by (Tomasi and Volkow 2012), albeit they used local FC density on young women and men with an age range of 18–30 years. Therefore it may represent a long standing difference between men and women at the level of these brain areas.

This study has limitations: The sample size is still relatively small for global generalization and we lack longitudinal data to monitor the progression from cognitive, structural, and FC perspectives. There are more women than men in the present sample, underscoring need for replication in samples with increased numbers of men. We included amyloid positive subjects in our definition of cognitively healthy subjects. Amyloid status was determined not to affect our current conclusions; there is a need for replication in a large sample of subjects without brain amyloid burden. The research has the strengths of a relatively large sample for a study incorporating fMRI in healthy elderly participants and use of global, regional, and traditional FC metrics of resting-state brain networks. The studies focus on cognitively healthy aging allowing observation of sex differences as part of the normal aging process, not as a part of neurodegeneration.

In conclusion, our study shows that functional brain-based differences between men and women are present in cognitively healthy aging, and these sex differences may have implications for understanding sex differences in pathological aging, including AD. Future studies that integrate a longitudinal approach with analyses of correlation/anticorrelation between different networks and different strategies of adaptation between men and women will help clarify the basis of these sex disparities.

## Funding

This research project was supported by the NIH (grant nos. 1RF1AG071566 and 5P20GM109025), Cleveland Clinic Keep Memory Alive Young Investigator Award, a private grant from Stacie and Chuck Matthewson, a private grant from Peter and Angela Dal Pezzo, and a private grant from Lynn and William Weidner. Data collection and sharing for this study was funded by the Alzheimer’s Disease Neuroimaging Initiative (ADNI) (National Institutes of Health Grant U01 AG024904) and DOD ADNI (Department of Defense award number W81XWH-12-2-0012) and Human Connectome Project. HCP funding was provided by the National Institute of Dental and Craniofacial Research (NIDCR), the National Institute of Mental Health (NIMH), and the National Institute of Neurological Disorders and Stroke (NINDS). ADNI is funded by the National Institute on Aging, the National Institute of Biomedical Imaging and Bioengineering, and through generous contributions from the following: AbbVie, Alzheimer’s Association; Alzheimer’s Drug Discovery Foundation; Araclon Biotech; BioClinica, Inc.; Biogen; Bristol-Myers Squibb Company; CereSpir, Inc.; Cogstate; Eisai Inc.; Elan Pharmaceuticals, Inc.; Eli Lilly and Company; EuroImmun; F. Hoffmann-La Roche Ltd and its affiliated company Genentech, Inc.; Fujirebio; GE Healthcare; IXICO Ltd; Janssen Alzheimer Immunotherapy Research & Development, LLC.; Johnson &Johnson Pharmaceutical Research & Development LLC.; Lumosity; Lundbeck; Merck & Co., Inc.; Meso Scale Diagnostics, LLC.; NeuroRx Research; Neurotrack Technologies; Novartis Pharmaceuticals Corporation; Pfizer Inc.; Piramal Imaging; Servier; Takeda Pharmaceutical Company; and Transition Therapeutics. The Canadian Institutes of Health Research is providing funds to support ADNI clinical sites in Canada. Private sector contributions are facilitated by the Foundation for the National Institutes of Health (www.fnih.org). The grantee organization is the Northern California Institute for Research and Education, and the study is coordinated by the Alzheimer’s Therapeutic Research Institute at the University of Southern California. ADNI data are disseminated by the Laboratory for Neuro Imaging at the University of Southern California.

*Conflict of interest statement*: The authors declare that there is no conflict of interest.

## Glossary

AD: Alzheimer disease; DN: Default Network; MCI: mild cognitive impairment; APOE4: apolipoprotein epsilon 4; FC: functional connectivity; ADAS-Cog: Alzheimer’s Disease Assessment Scale-Cognitive; RAVLT: Rey Auditory Verbal Learning Test; SUVR: standard uptake value ratio; fMRI: functional magnetic resonance imaging.

## Supplementary Material

CCC_Suppl_Tab_1_tgac023

CCC_Suppl_Tab_2_tgac023

CCC_Suppl_Fig_1-2_tgac023

## References

[ref1] Achard S , Bullmore E. Efficiency and cost of economical brain functional networks. PLoS Comput Biol. 2007:3(2):e17. 10.1371/journal.pcbi.0030017. 17274684 PMC1794324

[ref2] Alzheimer’s Association . 2021 Alzheimer’s disease facts and figures special report race, ethnicity and Alzheimer’s in America. Alzheimers Dement. 2021:17(3):327–406. 33756057 10.1002/alz.12328

[ref3] Biswal BB , Mennes M, Zuo XN, Gohel S, Kelly C, Smith SM, Beckmann CF, Adelstein JS, Buckner RL, Colcombe S, et al. Toward discovery science of human brain function. Proc Natl Acad Sci U S A. 2010:107(10):4734–4739. 10.1073/pnas.0911855107. 20176931 PMC2842060

[ref4] Brier M , Thomas JB, Snyder AZ, Benzinger TL, Zhang D, Raichle ME, Holtzman DM, Morris JC, Ances BM. Loss of intranetwork and internetwork resting state functional connections with Alzheimer's disease progression. J Neurosci. 2012:32(26):8890–8899. 10.1523/JNEUROSCI.5698-11.2012. 22745490 PMC3458508

[ref5] Buckner RL , Carroll DC. Self-projection and the brain. Trends Cogn Sci. 2007:11(2):49–57. 10.1016/j.tics.2006.11.004. 17188554

[ref6] Bullmore E , Sporns O. Complex brain networks: graph theoretical analysis of structural and functional systems. Nat Rev Neurosci. 2009:10(3):186–198. 10.1038/nrn2575. 19190637

[ref7] Ceci SJ , Williams WM, Barnett SM. Women’s underrepresentation in science: sociocultural and biological considerations. Psychol Bull. 2009:135(2):218–261. 10.1037/a0014412. 19254079

[ref8] Christoff K , Irving ZC, Fox KC, Spreng RN, Andrews-Hanna JR. Mind-wandering as spontaneous thought: a dynamic framework. Nat Rev Neurosci. 2016:17(11):718–731. 10.1038/nrn.2016.113. 27654862

[ref9] Cieri F , Esposito R. Neuroaging through the lens of the resting state networks. Biomed Res Int. 2018:2018:1–10. 10.1155/2018/5080981. PMC5820564 29568755

[ref10] Cieri F , Esposito R. Psychoanalysis and neuroscience: the bridge between mind and brain. Front Psychol. 2019:10:1790. 10.3389/fpsyg.2019.01983. 31555159 PMC6724748

[ref11] Cieri F , Cera N, Griffa A, Mantini D, Esposito R. Editorial: dynamic functioning of resting state networks in physiological and pathological conditions. Front Neurosci. 2020:14(December):1–6. 10.3389/fnins.2020.624401. 33390900 PMC7772206

[ref12] Cieri F , Zhuang X, Caldwell JZK, Cordes D. Brain entropy during aging through a free energy principle approach. Front Hum Neurosci. 2021a:15(March):1–19. 10.3389/fnhum.2021.647513. PMC8019811 33828471

[ref13] Cieri F , Yang Z, Cordes D, Caldwell JZK. Sex differences of brain functional topography revealed in normal aging and Alzheimer’s disease cohort. J Alzheimers Dis. 2021b:80(3):979–984. 10.3233/JAD-201596. 33612547 PMC8793667

[ref14] Contreras JA , Avena-Koenigsberger A, Risacher SL, West JD, Tallman E, McDonald BC, Farlow MR, Apostolova LG, Goñi J, Dzemidzic M, et al. Resting state network modularity along the prodromal late onset Alzheimer’s disease continuum. NeuroImage: Clinical. 2019:22(January):101687. 10.1016/j.nicl.2019.101687. 30710872 PMC6357852

[ref14a] Delbeuck X, Van der Linden M, Collette F. Alzheimer’s disease as a disconnection syndrome? Neuropsychol Rev. 2003:13:79–92. 10.1023/a:1023832305702. 12887040

[ref15] Esposito R , Cieri F, Chiacchiaretta P, Cera N, Lauriola M, di Giannantonio M, Tartaro A, Ferretti A. Modifications in resting state functional anticorrelation between default mode network and dorsal attention network: comparison among young adults, healthy elders and mild cognitive impairment patients. Brain Imaging Behav. 2018:12(1):127–141. 10.1007/s11682-017-9686-y. 28176262

[ref16] Fox MD , Raichle ME. Spontaneous fluctuations in brain activity observed with functional magnetic resonance imaging. Nat Rev Neurosci. 2007:8(9):700–711. 10.1038/nrn2201. 17704812

[ref17] Geerligs L , Renken RJ, Saliasi E, Maurits NM, Lorist MM. A brain-wide study of age-related changes in functional connectivity. Cereb Cortex. 2015:25(7):1987–1999. 10.1093/cercor/bhu012. 24532319

[ref17a] Geschwind N. Disconnexion syndromes in animals and man. Brain. 1965:88:237–294. 10.1093/brain/88.2.237. 5318481

[ref18] Gong G , Rosa-Neto P, Carbonell F, Chen ZJ, He Y, Evans AC. Age- and gender-related differences in the cortical anatomical network. J Neurosci. 2009:29(50):15684–15693. 10.1523/JNEUROSCI.2308-09.2009. 20016083 PMC2831804

[ref19] Grady C . The cognitive neuroscience of ageing (trends in neurocognitive aging). Nat Rev Neurosci. 2012:13(7):491–505. 10.1038/nrn3256. 22714020 PMC3800175

[ref20] Grady C , Sarraf S, Saverino C, Campbell K. Age differences in the functional interactions among the default, frontoparietal control, and dorsal attention networks. Neurobiol Aging. 2016:41:159–172. 10.1016/j.neurobiolaging.2016.02.020. 27103529

[ref21] Griffa A , Van Den Heuvel MP. Rich-club neurocircuitry: function, evolution, and vulnerability. Dialogues Clin Neurosci. 2018:20(2):121–132. 10.31887/dcns.2018.20.2/agriffa. 30250389 PMC6136122

[ref22] Griffa A , Baumann PS, Thiran JP, Hagmann P. Structural connectomics in brain diseases. NeuroImage. 2013:80:515–526. 10.1016/j.neuroimage.2013.04.056. 23623973

[ref23] Gur RC , Gur RE. Complementarity of sex differences in brain and behavior: from laterality to multimodal neuroimaging. J Neurosci Res. 2017:95(1–2):189–199. 10.1002/jnr.23830. 27870413 PMC5129843

[ref24] Hallett M , de Haan W, Deco G, Dengler R, Di Iorio R, Gallea C, Gerloff C, Grefkes C, Helmich RC, Kringelbach ML, et al. Human brain connectivity: clinical applications for clinical neurophysiology. Clin Neurophysiol. 2020:131(7):1621–1651. 10.1016/j.clinph.2020.03.031 Epub 2020 Apr 21. PMID: 32417703. 32417703

[ref27] Latora V , Marchiori M. Efficient behavior of small-world networks. Phys Rev Lett. 2001:87(19):198701. 10.1103/PhysRevLett.87.198701. 11690461

[ref28] Masuda N , Sakaki M, Ezaki T, Watanabe T. Clustering coefficients for correlation networks. Front Neuroinform. 2018:12(March):1–13. 10.3389/fninf.2018.00007. 29599714 PMC5863042

[ref29] Meunier D , Achard S, Morcom A, Bullmore E. Age-related changes in modular organization of human brain functional networks. NeuroImage. 2009:44(3):715–723. 10.1016/j.neuroimage.2008.09.062. 19027073

[ref30] Miller DI , Halpern DF. The new science of cognitive sex differences. Trends Cogn Sci. 2014:18(1):37–45. 10.1016/j.tics.2013.10.011. 24246136

[ref31] Raichle ME , MacLeod AM, Snyder AZ, Powers WJ, Gusnard DA, Shulman GL. A default mode of brain function. Proc Natl Acad Sci U S A. 2001:98(2):676–682. 10.1073/pnas.98.2.676. 11209064 PMC14647

[ref32] Ritchie SJ , Cox SR, Shen X, Lombardo MV, Reus LM, Alloza C, Harris MA, Alderson HL, Hunter S, Neilson E, et al. Sex differences in the adult human brain: evidence from 5216 UK biobank participants. Cereb Cortex. 2018:28(8):2959–2975. 10.1093/cercor/bhy109. 29771288 PMC6041980

[ref33] Rolls ET , Joliot M, Tzourio-Mazoyer N. Implementation of a new parcellation of the orbitofrontal cortex in the automated anatomical labeling atlas. NeuroImage. 2015:122:1–5. 10.1016/j.neuroimage.2015.07.075. 26241684

[ref34] Sala-Llonch R , Peña-Gómez C, Arenaza-Urquijo EM, Vidal-Piñeiro D, Bargalló N, Junqué C, Bartrés-Faz D. Brain connectivity during resting state and subsequent working memory task predicts behavioural performance. Cortex. 2012:48(9):1187–1196. 10.1016/j.cortex.2011.07.006. 21872853

[ref35] Simon HA . The architecture of complexity. The Roots of Logistics. 2012:106(6):335–361. 10.1007/978-3-642-27922-5_23.

[ref36] Stam CJ , Jones BF, Nolte G, Breakspear M, Scheltens P. Small-world networks and functional connectivity in Alzheimer’s disease. Cereb Cortex. 2007:17(1):92–99. 10.1093/cercor/bhj127. 16452642

[ref37] Stevens AA , Tappon SC, Garg A, Fair DA. Functional brain network modularity captures inter- and intra-individual variation in working memory capacity. PLoS One. 2012:7(1):e30468. 10.1371/journal.pone.0030468. 22276205 PMC3262818

[ref38] Supekar K , Menon V, Rubin D, Musen M, Greicius MD. Network analysis of intrinsic functional brain connectivity in Alzheimer’s disease. PLoS Comput Biol. 2008:4(6). 10.1371/journal.pcbi.1000100. PMC2435273 18584043

[ref39] Tomasi D , Volkow ND. Gender differences in brain functional connectivity density. Hum Brain Mapp. 2012:33(4):849–860. 10.1002/hbm.21252. 21425398 PMC3250567

[ref40] Tononi G , Sporns O, Edelman GM. A measure for brain complexity: relating functional segregation and integration in the nervous system. Proc Natl Acad Sci U S A. 1994:91(11):5033–5037. 10.1073/pnas.91.11.5033. 8197179 PMC43925

[ref25] van den Heuvel MP , Sporns O. Rich-club organization of the human connectome. J Neurosci. 2011:31(44):15775–15786. 10.1523/JNEUROSCI.3539-11.2011. 22049421 PMC6623027

[ref26] van den Heuvel MP , Sporns O. Network hubs in the human brain. Trends Cogn Sci. 2013:17(12):683–696. 10.1016/j.tics.2013.09.012. 24231140

[ref41] Wang J , Zuo X, Dai Z, Xia M, Zhao Z, Zhao X, Jia J, Han Y, He Y. Disrupted functional brain connectome in individuals at risk for Alzheimer’s disease. Biol Psychiatry. 2013:73(5):472–481. 10.1016/j.biopsych.2012.03.026. 22537793

[ref42] Wang J , Wang X, Xia M, Liao X, Evans A, He Y. GRETNA: a graph theoretical network analysis toolbox for imaging connectomics. Front Hum Neurosci. 2015:9(JUNE):1–16. 10.3389/fnhum.2015.00386. 26175682 PMC4485071

[ref43] Watts DJ , Strogatz SH. Collective_dynamics_small_world_networks. 1998. Nature. 1998:393(6684):440–442. 10.1038/30918. 9623998

[ref44] Weis S , Patil KR, Hoffstaedter F, Nostro A, Yeo BTT, Eickhoff SB. Sex classification by resting state brain connectivity. Cereb Cortex. 2020:30(2):824–835. 10.1093/cercor/bhz129. 31251328 PMC7444737

[ref45] Yang Z , Zhuang X, Sreenivasan K, Mishra V, Cordes D, Alzheimer's Disease Neuroimaging Initiative. Disentangling time series between brain tissues improves fMRI data quality using a time-dependent deep neural network. NeuroImage. 2020:223(July):117340. 10.1016/j.neuroimage.2020.117340. 32898682 PMC7792822

[ref46] Yang Z , Caldwell JZK, Cummings JL, Ritter A, Kinney JW, Cordes D, Alzheimer's Disease Neuroimaging Initiative (ADNI). Sex modulates the pathological aging effect on caudate functional connectivity in mild cognitive impairment. Front Psych. 2022:13:804168. 10.3389/fpsyt.2022.804168. PMC9037326 35479489

[ref47] Yao Z , Zhang Y, Lin L, Zhou Y, Xu C, Jiang T, the Alzheimer's Disease Neuroimaging Initiative. Abnormal cortical networks in mild cognitive impairment and alzheimer’s disease. PLoS Comput Biol. 2010:6(11):e1001006. 10.1371/journal.pcbi.1001006. 21124954 PMC2987916

[ref48] Zalesky A , Fornito A, Bullmore ET. Network-based statistic: identifying differences in brain networks. NeuroImage. 2010:53(4):1197–1207. 10.1016/j.neuroimage.2010.06.041. 20600983

