# Supplementary material for: Brain functional topology differs by sex in cognitively normal older adults
Source: Cereb Cortex Commun. 2022 Jun 27;3(3):tgac023. doi: 10.1093/texcom/tgac023 (PMC9252274; doi:10.1093/texcom/tgac023)
Supplement: CCC_Suppl_Fig_1-2_tgac023 [file ccc_suppl_fig_1-2_tgac023.docx]

**Supplementary Figure 1**


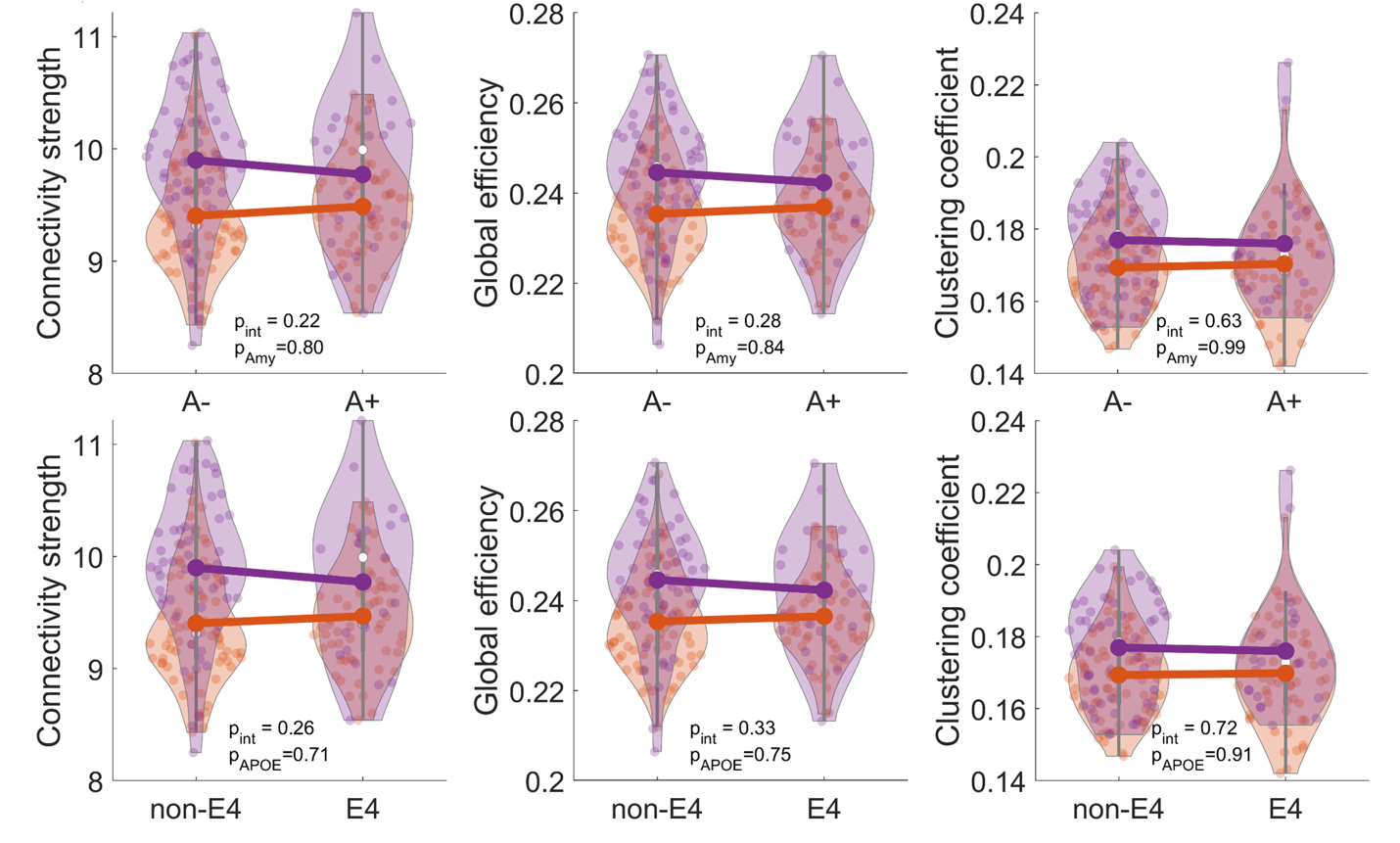


**Supplementary Figure 1. Role of APOE and amyloid status on global network metrics. [a] Violin plots for APOE E4 and non-E4 women and men. The significance of APOE status [*p*_APOE_] and its interaction with sex [*p*_int_] on global network metrics were derived from two-way analysis of variance. The mean values for the E4 carriers and non-E4 carriers were connected with purple and orange lines for men and women, respectively. [b] Violin plots for amyloid positive and amyloid negative women and men. The significance of amyloid status [*p*_APOE_] and its interaction with sex [*p*_int_] were derived from two-way analysis of variance. Neither the main effect nor the interaction effect with sex of APOE and amyloid status were significant. The mean values for the amyloid negative [A-] and amyloid positive [A+] participants were connected with purple and orange lines for men and women, respectively.**

**Supplementary Figure 2**

**
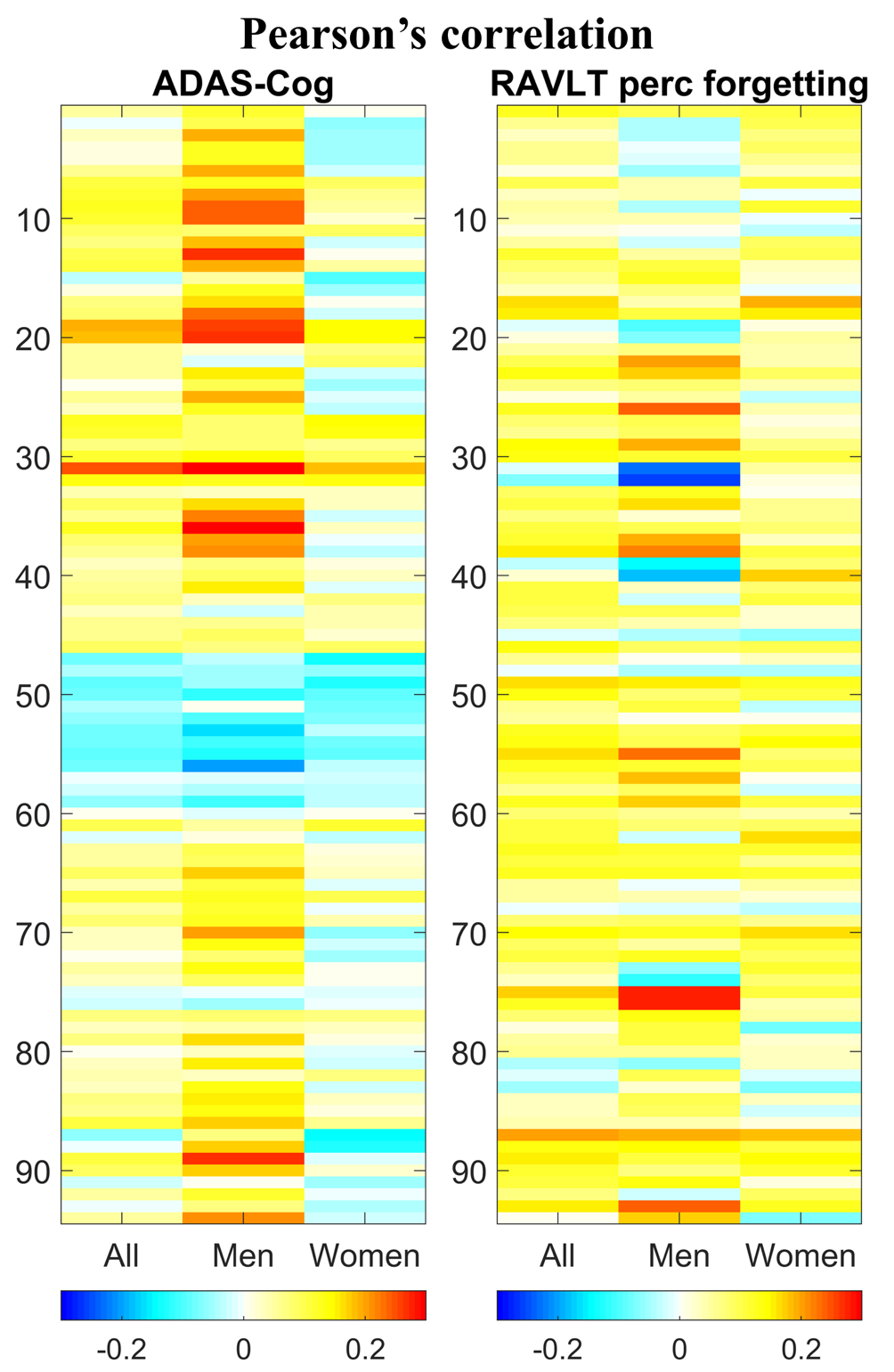
**

**Supplementary Figure 2. Association of regional clustering coefficients with ADAS-Cog and RAVLT percent forgetting scores.**
